# Computing the size and number of neuronal clusters in local circuits

**DOI:** 10.3389/fnana.2013.00001

**Published:** 2013-02-19

**Authors:** Rodrigo Perin, Martin Telefont, Henry Markram

**Affiliations:** Brain Mind Institute, Ecole Polytechnique Fédérale de LausanneLausanne, Switzerland

**Keywords:** data driven modeling, neuronal assemblies, layer 5 pyramidal cell, layer 2 pyramidal cell, clustering

## Abstract

The organization of connectivity in neuronal networks is fundamental to understanding the activity and function of neural networks and information processing in the brain. Recent studies show that the neocortex is not only organized in columns and layers but also, within these, into synaptically connected clusters of neurons (Ko et al., 2011; Perin et al., 2011). The recently discovered common neighbor rule, according to which the probability of any two neurons being synaptically connected grows with the number of their common neighbors, is an organizing principle for this local clustering. Here we investigated the theoretical constraints for how the spatial extent of neuronal axonal and dendritic arborization, heretofore described by morphological reach, the density of neurons and the size of the network determine cluster size and numbers within neural networks constructed according to the common neighbor rule. In the formulation we developed, morphological reach, cell density, and network size are sufficient to estimate how many neurons, on average, occur in a cluster and how many clusters exist in a given network. We find that cluster sizes do not grow indefinitely as network parameters increase, but tend to characteristic limiting values.

## Introduction

Network theory is increasingly applied to better understand the principles of how neurons are interconnected and hence to unravel the networking and topologic mysteries of the brain (Honey et al., 2007, 2010; Sporns et al., 2007; Sporns, 2010). Much of this work has been focused on macroscopic network principles such as those connecting brain regions. Increased interest in mesoscopic neuroanatomical connectivity also grew considerably over the years (Bohland et al., 2009). Recently, the discovery of clusters of synaptically connected neurons has opened up investigations of the microscopic network principles. The expression of a number of cellular level motifs of synaptic connectivity was first reported by Song et al. (2005). Subsequent publications on the rat (Perin et al., 2011) and mouse (Ko et al., 2011), hint at a theme shared by different species.

**Figure 1:**
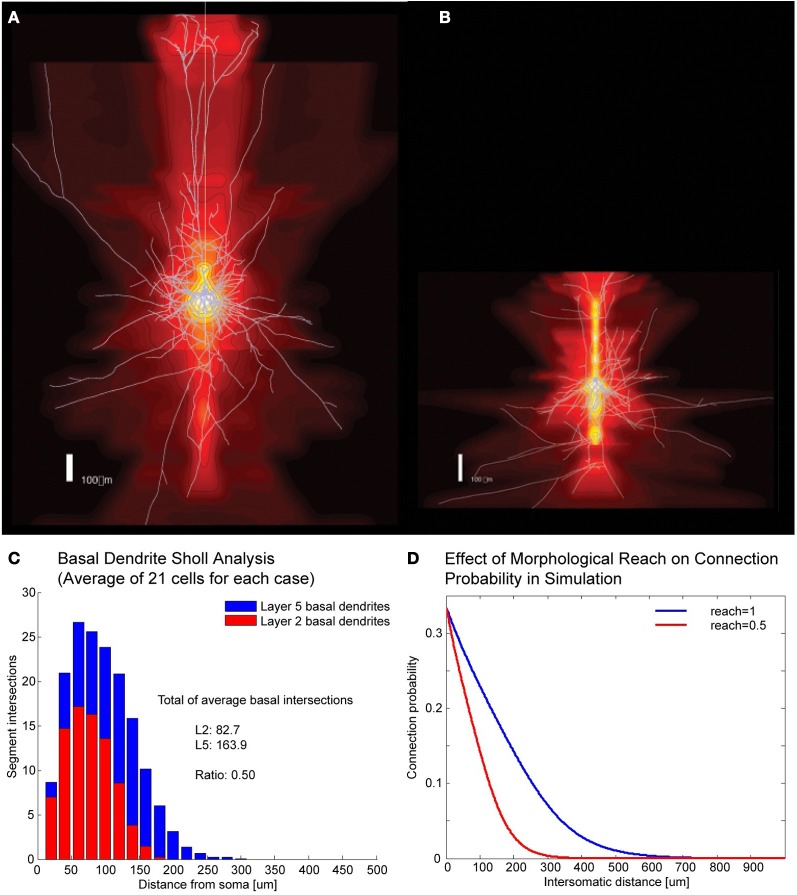
**Correlates of different cell types. (A,B)** Example of Layer V Pyramidal Cell and Layer II/III Pyramidal Cell morphologies, respectively, superposed on intensity maps representing the average density of morphological processes around the soma. **(C)** Sholl analysis of the basal dendrites of Layer V and Layer II/III. **(D)** Illustration of how morphological reach is used to modify connection probabilities as a function of inter-somatic distance in simulations.

A number of cortical networks that have been described are based on cells with large somata, which are easy to distinguish in electrophysiological experiments. The cell of choice in the neocortex has been the large thick tufted layer 5 pyramidal cell (TTL5-PCs) which, thanks to its size and shape, can be isolated and recorded reliably (Markram et al., 1997). In a multi-electrode patch-clamp setup it is possible to record from many such cells simultaneously, determine their synaptic connectivity and, after staining, also obtain their morphological properties. In order to simulate different expanses of axonal and dendritic arborization (Figures 1A,B) we developed the concept of “morphological reach,” denoted *r*, a proportionality factor applied to the decay in connection probability as a function of distance. Our reference, the TTL5-PC (Oberlaender et al., 2011), is assigned a morphological reach of 1. Other cell types, in our simulations, are assigned proportional morphological reaches indicative of the extent of their basal dendritic arborizations as measured using the sum of all branch intersections in Sholl Analyses (Figures 1C,D). TTL5 pyramidal cells have broad dendritic arborization (Markram et al., 1997) and form clusters that may constitute elementary units of information processing within a brain region (medium/regional projections) and between brain regions (long/interregional projections). In previous work we identified a rule, the common neighbor rule, governing the connectivity of these groups of neurons (Perin et al., 2011). The common neighbor rule describes a directly proportional relationship between the connection probability between any two neurons and the number of other neurons in the network connected to both neurons in this pair. In this case the term connected indicates neurons that project to as well as neurons receiving synaptic appositions from the neurons in the pair. When applied, the common neighbor rule produces complex clustered networks that lie between completely random networks (Erdös–Reni-type) (Erdõs and Rényi, 1960) and highly clustered networks with hubs (Barabási-type) (Barabási and Albert, 1999) resembling more closely a Watts-Strogatz-type network (Watts and Strogatz, 1998). A key distinguishing feature between macro-clustering in the brain (between brain regions) and micro-clustering (between neurons of a local microcircuit) is that there are no hubs at the micro (local) network level within a given cell-type. In other words, each neuron of a given type makes contact with and receives contacts from about the same number of neurons of the same type—in fact a pre-requisite for the occurrence of hubs is that the number of boutons and spines per neuron within the population span some orders of magnitude so that the degree of connectivity might vary accordingly. This form of clustering is quite exceptional amongst biological and social networks in that it contains no hubs and has a degree of separation less than 2.

**Figure A1:**
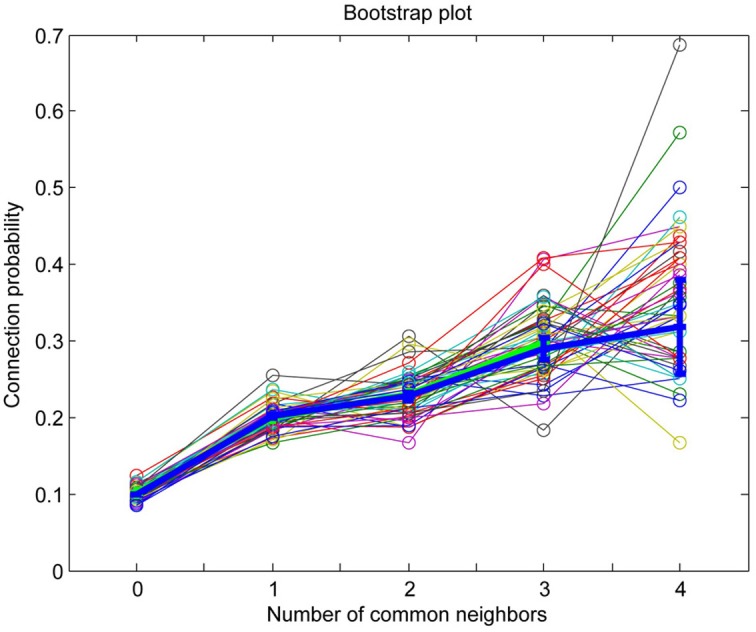
**MatLab bootstrap analysis of connection probability as a function of the number of common neighbors (thin lines in various colors) with original data mean and standard error of the mean superposed for reference (thick blue line)**.

**Figure A2:**
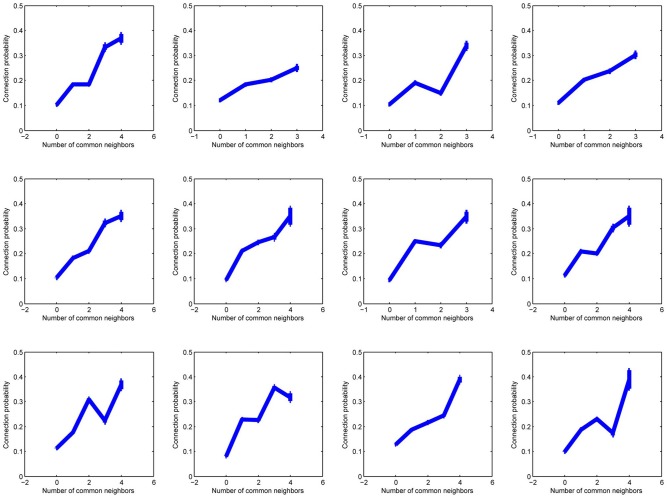
**Twelve plots of connection probability as a function of the number of common neighbors in subsets of 20 experiments, each experiment from a different individual**.

Since the common neighbor based clustering applies similarly across individuals of the same species, the neuronal groups formed would be quite similar. Bootstrap analysis, in which different subsets of the connectivity data originating from different individuals are used, consistently captures the common neighbor rule (Figures A1, A2). This has led to the suggestion that these clusters are in fact innate (genetically preprogrammed to develop) holding innate knowledge of elementary forms of information processing. These posited innate groups likely require some general activity, rather than experience-dependent activity, during development to form.

It may therefore be of special interest to understand the sizes and numbers of such clusters in a brain region. We therefore constructed networks by applying the common neighbor rule and derived the relationships between cluster sizes and numbers as a function of morphological reach, neuronal density, and network size.

## Methods

**Figure 2:**
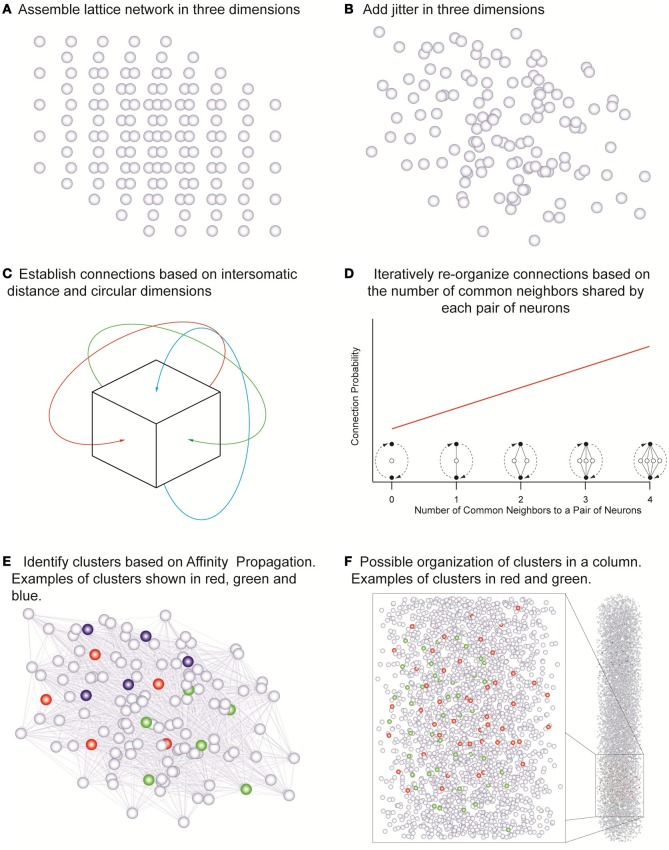
**The network assembly and reorganization of connections. (A)** Initial lattice arrangement of somas. **(B)** Jitter added in three dimensions to the lattice arrangement. **(C)** Connections assigned according to intersomatic distance probability profiles in circular dimensions in order to eliminate boundary effects. **(D)** Iterative reorganization of connections based on numbers of common neighbors. **(E)** Cluster identification based on affinity propagation. **(F)** Possible organization of clusters in a column with examples of clusters sharing the same space.

Point neurons were placed in a cubic tridimensional lattice arrangement with regular spacing that assumed the values of 18, 19, 21, 23, 25, 28, and 36 μm in different simulations, yielding the corresponding cell densities of 171470, 145790, 107980, 82190, 64000, 45554, and 21433 neurons/mm^3^ (Figure 2A) covering the range of experimentally observed densities in a neocortical column (Meyer et al., 2010). Uniformly random jitter was then added to each position in three dimensions with amplitude equal to the grid spacing between neurons (Figure 2B). Since point neurons are used no compensation for extreme proximity between neurons is applied. The simulations used network sizes of 512, 1000, 1728, 3375, 4096, and 5832 cells in cubic arrangements. Each of the three dimensions in this model was made circular so that neurons close to one edge were considered to be close to neurons on the opposite edge of the network (Figure 2C). Edge effects were countered in this way and every neuron was exposed to a similar number of neighbors and could potentially form the same number of connections.

**Figure A3:**
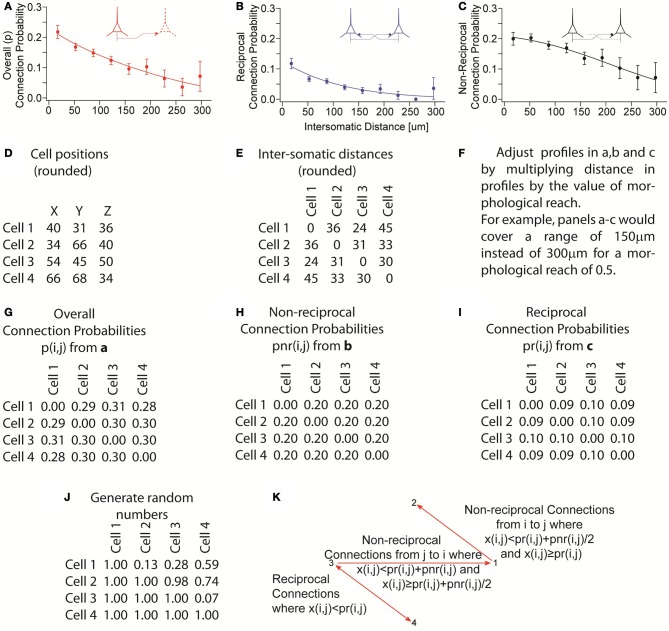
**Process illustrating assembly of pseudo-random networks that respect the overall connection probability as a function of distance as well as the non-reciprocal and reciprocal connection probabilities as a function of distance. (A–C)** Experimentally observed connection probability profiles and fitted functions (sigmoids). **(D)** Example of cell positions generated by grid plus random jitter. **(E)** Calculated intersomatic distances from **D**. **(F)** Apply morphological reach as a factor in the connection probability profile. **(G–I)** Connection probabilities, given a morphological reach of 1, used for the remainder of the example. **(J)** Generate random numbers *x*(*i*, *j*) once for each pair of neurons *i* and *j*. **(K)** Determine presence and direction of connections based on random numbers that were generated. Reciprocal connections occur where *x*(*i*, *j*) < pr(*i*, *j*). Non-reciprocal connections from neuron *i* to neuron *j* occur where *x*(*i*, *j*) ≥ pr(*i*, *j*) and *x*(*i*, *j*) < pr(*i*, *j*) + pnr(*i*, *j*)/2. Non-reciprocal connections occur from neuron *j* to neuron *i* where *x*(*i*, *j*) ≥ pr(*i*, *j*) + pnr(*i*, *j*)/2 and *x*(*i*, *j*) < pr(*i*, *j*) + pnr(*i*, *j*). No connections are formed when *x*(*i*, *j*) ≥ pr(*i*, *j*) + pnr(*i*, *j*). The overall probability of connection *p* always respects its distance profile and the equation *p*(*i*, *j*) = pr(*i*, *j*) + pnr(*i*, *j*)/2.

**Figure A4:**
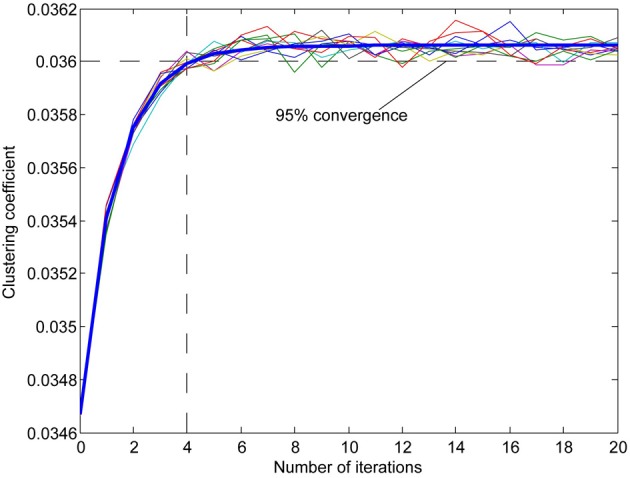
**Examples of the effect of reorganization on clustering coefficient of a network (multiple thin lines) with a fitted exponential curve (thick blue line).** Since the computation of the clustering coefficient is time-consuming we used the number of iterations that best approximated 95% percent convergence to the value projected after infinite iterations (from fitted data). The clustering coefficient is the measure of the probability of connection among each neuron's neighbors calculated for each neuron in the network.

In order to investigate the influence of morphology on clustering, the morphological reach of TTL5 pyramidal cells was multiplied by factors of 0.5, 0.667, 0.8, 1, 1.333, and 2 in networks containing 4096 neurons. We defined a neuron's neighbors as those cells that form synaptic connections onto or that receive synaptic connections from the neuron in question. In our model, the small world clustering of layer 5 pyramidal cells is made directly proportional to the number of common neighbors (Figure 2D) (Perin et al., 2011). To achieve this goal the connections in the initial network were assigned pseudo-randomly according to inter-somatic distance profiles observed experimentally (Perin et al., 2011) (see also Figure A3). While morphological reconstructions indicate a degree of anisotropy in process arborization such observations indicate that a purely distance-based connection probability function still can capture the trend in connectivity patterns. In the simulations where we were interested in simulating different arborizations we linearly extended or shortened such profiles by dividing the actual distance supplied to these profiles by the “morphological reach” factor. The initial connectivity was then modified according to the number of common neighbors. The pair of neurons sharing the maximal number of common neighbors was assigned a probability of connection of 1. Pairs of neurons sharing no common neighbors we assigned a connection probability of 0. Pairs of neurons sharing intermediate numbers of common neighbors were assigned linearly interpolated connection probability values. This process was iterated until the clustering coefficient of the whole network no longer increased (see Figure A4). The observed clusters consist of a few dozen neurons typically distributed 100–150 μm apart. Based on this study (Perin et al., 2011) we decided to investigate the effects of different network sizes and cell densities as well as the impact of different extents of morphological arborization on the clustering properties of networks. Clusters were identified using the affinity propagation algorithm (Frey and Dueck, 2007) at the end of the simulation and providing the number of common neighbors as the similarity measure (Figure 2E). Similarity was defined exclusively for connected neuron pairs, being set to zero in pairs that were not connected in any way.

Sets of 10 simulations were performed for each parameter combination. The average numbers of clusters and average cluster sizes were plotted and the data points were fitted with surface fits using the MatLab curve fitting tool.

## Results

The goal of our modeling was to better understand the relationship between the different network parameters and the network clustering properties. Six basic relationships were investigated by varying three parameters—network size, cell density, and morphological reach—and analyzing the effects on the two clustering properties—the average number of clusters and the average number of cells contained in clusters (cluster size). From these results we also calculated the number of clusters per unit volume.

### Network size and density

**Figure 3:**
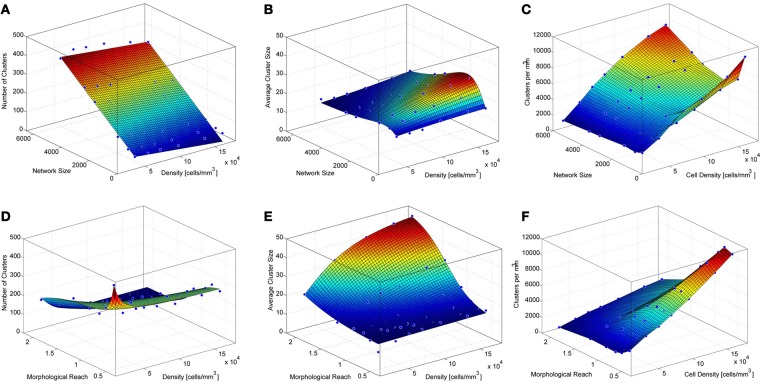
**Properties of simulated cortical network clustering. (A)** Average number of clusters observed in the different simulations as a function of network size and density. Each data point corresponds to the average of 10 simulations. **(B)** Average cluster size for the same simulations show in **A**. **(C)** Number of clusters per mm^3^ calculated from **A** and **B**. **(D)** Average number of clusters observed in the different simulations as a function of network density and morphological reach. Each green data point corresponds to the average of 10 simulations. **(E)** Average cluster size for the same simulations show in **D**. **(F)** Number of clusters per mm^3^ calculated from **D** and **E**.

To investigate the impact of network size on the number and size of clusters we constructed networks ranging from 512 to 5832 neurons. These networks were based on TTL5 Pyramidal Cells and ranged in density from 45554 to 455170 cells/mm^3^. Networks containing different numbers of neurons display different clustering properties. A relatively simple linear relationship exists between the network size and the number of clusters. Simply put, larger networks fit more clusters of similar size (Figure 3A) as can observed in the curve that fits the surface that describes the number of cluster as a function of networks size and density:

$$NC=21.54-\frac{3.90}{10000}d+\frac{6.42}{100}s$$

where *NC* stands for the number of clusters, *s* for network size in number of cells and *d* for network density in cells/mm^3^.

A mild decay in the number of clusters occurs as the network density increases. This is because higher densities lead to larger average cluster sizes (Figure 3B). Cluster size as a function of network size follows a lognormal profile, first increasing then decreasing. This is a particular feature of networks without boundaries. The fitted surface for cluster sizes as a function of network size and density can be expressed as

$$CS=13.23+\frac{d}{10000}e^{-{\left(\log\left(\frac{s}{1300+\frac{3d}{1000}}\right)\;\right)}^{2}}$$

where *CS* stands for cluster size, *s* for network size in number of cells and *d* for network density in cells/mm^3^. Goodness of fit for the resulting surfaces according to Adjusted R-square values were 0.97 for average number of clusters as a function of network density and size, 0.95 for average cluster sizes as a function of network density and size. The number of clusters per unit volume grows with cell density and displays an inverted relationship to cluster size as a function of network size (Figure 3C).

### Network density and morphological reach

Morphological reach is a parameter that reflects the extent of neuronal arborization (Figures 1A,B). The more extensive the arborization the more connections a neuron is likely to form (Figure 1C). We attribute the value of 1 to the morphological reach characteristic of TTL5 Pyramidal Cells. Cells with more restricted arborization display, therefore, morphological reaches corresponding to some fractional value (Figure 1D).

**Figure A5:**
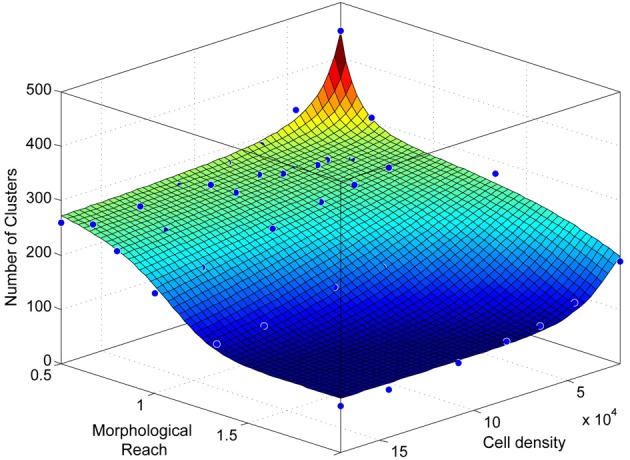
**Number of clusters as a function of network density and morphological reach as in Figure 3D, viewed from a different angle**.

The density of neurons in a local network, in the following simulations involving 4096 neurons, strongly influences the number of clusters formed, particularly very low densities which cause neurons to be more isolated thus yielding numerous clusters involving few neurons (Figures 3D, A5). As neuronal densities increase the number of clusters quickly becomes less dependent on this parameter reaching a plateau before decreasing again due to increasing cluster sizes (Figures 3D,E). The relationship between the number of clusters in a network and the morphological reach of the cells that form this network follows a similar trend, also forming a plateau before further decreasing.

$$NC=100.70+\frac{210.60}{1+e^{-(1.57-dr^{3}/1e5)/0.97}}+\frac{1.23e9}{{\left(dr^{3}\right)}^{2}}$$

where *NC* stands for the number of clusters, *r* for morphological reach and *d* for network density in cells/mm^3^. Morphological reach, similarly to density, has little effect on the number of clusters in a network except in the case of extremely low values, which lead to nearly isolated neurons (Figure 3D). Within the physiological range of 0.5–1, growth in morphological reach leads to approximately linear and relatively mild increases in cluster sizes. Increases in morphological reach above 1 tend to have larger effects, especially in the case of networks with large cell density but eventually tend to saturation at extreme values of both. Changes in reach and density led to different cluster sizes (Figure 3E) according to a sigmoidal:

$$CS=-13.78+\frac{62.37}{1+e^{\frac{8.36e4-dr^{3}}{3.23e5}}}$$

where *CS* stands for cluster size, *d* for network density in cells/mm^3^ and *r* for morphological reach. It is important to capture variations in morphological reach and cell density since the combination of both is necessary in order to estimate clustering properties.

Goodness of fit for the resulting surfaces according to Adjusted R-square values was 0.97 for average number of clusters as a function of network density and morphological reach, 0.98 for average cluster sizes as a function of network density and morphological reach. The number of clusters per unit volume grows approximately linearly with both cell density and morphological reach (Figure 3F).

## Discussion

The most abundant neuronal type in the mammal neocortex, the pyramidal cell can be found in five of the six neocortical layers and with varying spatial reach of their arbors. This cell retains a remarkable stereotypical shape from mice to man and varies mainly in the length of its axonal and dendritic arbors (Peters and Yilmaz, 1993). Thick tufted layer 5 pyramidal cells are among the largest neurons in the brain and provide the output from the neocortex to subcortical structures and distant brain regions to drive behavior (Morishima and Kawaguchi, 2006). We previously found that these pyramidal cells are networked according to a simple synaptic organization rule; neurons that share more common neighbors are also more likely to be connected (Perin et al., 2011). This rule results in stereotypical clusters of synaptically connected neurons and therefore do not seem to be uniquely shaped by experience. In fact, the synaptic weights of the connections between neurons in these clusters are also on average saturated, which is not ideal for acquired memory storage. We therefore propose that forming synaptic connections according to common neighbors is a pre-programmed rule that drives stereotypical neuronal clustering during development. These clusters may therefore express elementary units of innate knowledge that are combined in an experience-dependent manner to form acquired memories while preserving fundamental perceptual mechanisms. It is likely that at its origin, cluster formation may be linked to the fact that sister cells (i.e., cells that originated from the same progenitor radial glial cell) are more likely to develop strong electrical coupling which in turn favors chemical synapse formation (Yu et al., 2009, 2012). These preferentially connected sets of neurons may associate to form larger ensembles as those involved in orientation selectivity in the visual cortex (Ko et al., 2011).

The common neighbor rule also makes it possible to pre-specify the underlying synaptic connectivity in a network of such neurons even before learning rules come into play. The influence from common input leading to greater connection probabilities between neurons, which constituted the central mechanism explored in the current work, seems to apply not only for local but also long-range projections (Otsuka and Kawaguchi, 2008; Brown and Hestrin, 2009). The question we focused on, however, is how the structural features of neuronal arborization further influence the local synaptic connectivity organization. The common neighbor rule, which ensures that important features of synaptic organization are respected, was therefore imposed on the connectivity between neurons with different morphological reach and cell densities. From these networks we derived relationships between morphological reach, cell density and number of cells in a network, in order to determine how many neurons make up the clusters and how many clusters can potentially be formed under different conditions.

Experimental evidence from juvenile rat somatosensory cortex (Perin et al., 2011) as well as adult visual, (Ohki et al., 2005; Yoshimura et al., 2005) and auditory (Rothschild et al., 2010) and mouse frontal cortices (Otsuka and Kawaguchi, 2011) supports the occurrence of clusters of excitatory cells in Layers II/III through V, overlapped and interlaced in space, rather than tiled next to each other (Figures 2E,F). This not only allows the packing of many clusters of neurons in the same space but also enables monosynaptic connections between clusters to be adjusted and regulate the relationship between clusters.

Future investigations into this topic should also take into consideration the effects of network boundaries and interactions between different cell types, both constituting factors that further influence cluster formation.

### Conflict of interest statement

The authors declare that the research was conducted in the absence of any commercial or financial relationships that could be construed as a potential conflict of interest.

## References

[B1] Barabási A.-L. Albert R. (1999). Emergence of scaling in random networks. Science 286, 509–512 10.1126/science.286.5439.509 10521342

[B2] Bohland J. W. Wu C. Barbas H. Bokil H. Bota M. Breiter H. C. (2009). A proposal for a coordinated effort for the determination of brainwide neuroanatomical connectivity in model organisms at a mesoscopic scale. PLoS Comput. Biol. 5:e1000334 10.1371/journal.pcbi.1000334 19325892 PMC2655718

[B3] Brown S. P. Hestrin S. (2009). Intracortical circuits of pyramidal neurons reflect their long-range axonal targets. Nature 457, 1133–1136 10.1038/nature07658 19151698 PMC2727746

[B4] Erdõs P. Rényi A. (1960). On the evolution of random graphs. Publ. Math. Inst. Hung. Acad. Sci. 5, 17–61

[B5] Frey B. J. Dueck D. (2007). Clustering by passing messages between data points. Science 315, 972–976 10.1126/science.1136800 17218491

[B6] Honey C. J. Kötter R. Breakspear M. Sporns O. (2007). Network structure of cerebral cortex shapes functional connectivity on multiple time scales. Proc. Natl. Acad. Sci. U.S.A. 104, 10240–10245 10.1073/pnas.0701519104 17548818 PMC1891224

[B7] Honey C. J. Thivierge J.-P. Sporns O. (2010). Can structure predict function in the human brain? Neuroimage 52, 766–776 10.1016/j.neuroimage.2010.01.071 20116438

[B8] Ko H. Hofer S. B. Pichler B. Buchanan K. A. Sjöström P. J. Mrsic-Flogel T. D. (2011). Functional specificity of local synaptic connections in neocortical networks. Nature 473, 87–91 10.1038/nature09880 21478872 PMC3089591

[B9] Markram H. Lübke J. Frotscher M. Roth A. Sakmann B. (1997). Physiology and anatomy of synaptic connections between thick tufted pyramidal neurones in the developing rat neocortex. J. Physiol. 500, 409–440 9147328 10.1113/jphysiol.1997.sp022031 PMC1159394

[B10] Meyer H. S. Wimmer V. C. Oberlaender M. de Kock C. P. J. Sakmann B. Helmstaedter M. (2010). Number and laminar distribution of neurons in a thalamocortical projection column of rat vibrissal cortex. Cereb. Cortex 20, 2277–2286 10.1093/cercor/bhq067 20534784 PMC2936806

[B11] Morishima M. Kawaguchi Y. (2006). Recurrent connection patterns of corticostriatal pyramidal cells in frontal cortex. J. Neurosci. 26, 4394–4405 10.1523/JNEUROSCI.0252-06.2006 16624959 PMC6674016

[B12] Oberlaender M. Boudewijns Z. S. R. M. Kleele T. Mansvelder H. D. Sakmann B. de Kock C. P. J. (2011). Three-dimensional axon morphologies of individual layer 5 neurons indicate cell type-specific intracortical pathways for whisker motion and touch. Proc. Natl. Acad. Sci. U.S.A. 108, 4188–4193 10.1073/pnas.1100647108 21368112 PMC3053980

[B13] Ohki K. Chung S. Ch'ng Y. H. Kara P. Reid R. C. (2005). Functional imaging with cellular resolution reveals precise micro-architecture in visual cortex. Nature 433, 597–603 10.1038/nature03274 15660108

[B14] Otsuka T. Kawaguchi Y. (2008). Firing-pattern-dependent specificity of cortical excitatory feed-forward subnetworks. J. Neurosci. 28, 11186–11195 10.1523/JNEUROSCI.1921-08.2008 18971461 PMC6671518

[B15] Otsuka T. Kawaguchi Y. (2011). Cell diversity and connection specificity between callosal projection neurons in the frontal cortex. J. Neurosci. 31, 3862–3870 10.1523/JNEUROSCI.5795-10.2011 21389241 PMC6622807

[B16] Perin R. Berger T. K. Markram H. (2011). A synaptic organizing principle for cortical neuronal groups. Proc. Natl. Acad. Sci. U.S.A. 108, 5419–5424 10.1073/pnas.1016051108 21383177 PMC3069183

[B17] Peters A. Yilmaz E. (1993). Neuronal organization in area 17 of cat visual cortex. Cereb. Cortex 3, 49–68 10.1093/cercor/3.1.49 7679939

[B18] Rothschild G. Nelken I. Mizrahi A. (2010). Functional organization and population dynamics in the mouse primary auditory cortex. Nat. Neurosci. 13, 353–360 10.1038/nn.2484 20118927

[B19] Song S. Sjöström P. J. Reigl M. Nelson S. Chklovskii D. B. (2005). Highly nonrandom features of synaptic connectivity in local cortical circuits. PLoS Biol. 3:e68 10.1371/journal.pbio.0030068 15737062 PMC1054880

[B20] Sporns O. (2010). Networks of the Brain. Cambridge, MA: The MIT Press

[B21] Sporns O. Honey C. J. Kötter R. (2007). Identification and classification of hubs in brain networks. PLoS ONE 2:e1049 10.1371/journal.pone.0001049 17940613 PMC2013941

[B22] Watts D. J. Strogatz S. H. (1998). Collective dynamics of /‘small-world/’ networks. Nature 393, 440–442 10.1038/30918 9623998

[B23] Yoshimura Y. Dantzker J. L. M. Callaway E. M. (2005). Excitatory cortical neurons form fine-scale functional networks. Nature 433, 868–873 10.1038/nature03252 15729343

[B24] Yu Y.-C. Bultje R. S. Wang X. Shi S.-H. (2009). Specific synapses develop preferentially among sister excitatory neurons in the neocortex. Nature 458, 501–504 10.1038/nature07722 19204731 PMC2727717

[B25] Yu Y.-C. He S. Chen S. Fu Y. Brown K. N. Yao X.-H. (2012). Preferential electrical coupling regulates neocortical lineage-dependent microcircuit assembly. Nature 486, 113–117 10.1038/nature10958 22678291 PMC3599787

